# Experimental realization of chiral Landau levels in two-dimensional Dirac cone systems with inhomogeneous effective mass

**DOI:** 10.1038/s41377-023-01209-z

**Published:** 2023-07-04

**Authors:** Hongwei Jia, Mudi Wang, Shaojie Ma, Ruo-Yang Zhang, Jing Hu, Dongyang Wang, Che Ting Chan

**Affiliations:** 1 Department of Physics, the Hong Kong University of Science and Technology, Clear Water Bay, Kowloon, Hong Kong, China; 2 Institute for Advanced Study, the Hong Kong University of Science and Technology, Clear Water Bay, Kowloon, Hong Kong, China; 3 Department of Physics, University of Hong Kong, Hong Kong, China

**Keywords:** Photonic crystals, Optical physics

## Abstract

Chiral zeroth Landau levels are topologically protected bulk states. In particle physics and condensed matter physics, the chiral zeroth Landau level plays a significant role in breaking chiral symmetry and gives rise to the chiral anomaly. Previous experimental works on such chiral Landau levels are mainly based on three-dimensional Weyl degeneracies coupled with axial magnetic fields. Their realizations using two-dimensional Dirac point systems, being more promising for future applications, were never experimentally realized before. Here we propose an experimental scheme for realizing chiral Landau levels in a two-dimensional photonic system. By introducing an inhomogeneous effective mass through breaking local parity-inversion symmetries, a synthetic in-plane magnetic field is generated and coupled with the Dirac quasi-particles. Consequently, the zeroth-order chiral Landau levels can be induced, and the one-way propagation characteristics are experimentally observed. In addition, the robust transport of the chiral zeroth mode against defects in the system is also experimentally tested. Our system provides a new pathway for the realization of chiral Landau levels in two-dimensional Dirac cone systems, and may potentially be applied in device designs utilizing the chiral response and transport robustness.

## Abstract (web summary)

A 2D Dirac cone photonic system with inhomogeneous effective mass gives rise to an in-plane synthetic magnetic field, inducing chiral zeroth Landau levels with one-way propagative characteristics.

## Graphical abstract

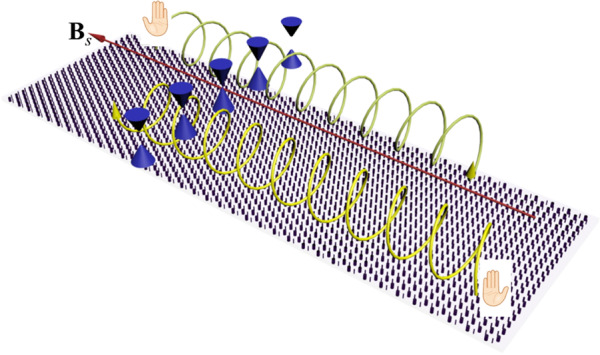

## Introduction

Linear degeneracies in band structures are singularities in momentum space. These singularities are the sources of topological characteristics in band theory. Typical examples are the Dirac and Weyl points^1–13^, which are linear crossings of two energy bands. They give rise to nontrivial topological invariants, which are associated with a variety of novel phenomena, such as topologically protected gapless surface states that connect two singular points with opposite chiralities^6,8–10,13^, and singular points with opposite topological charges can annihilate each other^1,11^. Since the dynamical equation describing the Weyl or Dirac cones has a form similar to that of relativistic Fermions in quantum field theory^12^, such systems are good platforms for emulating relativistic particles and observing the single particle behaviors. Recent advances in inhomogeneous modulations on unit cell morphologies successfully introduced synthetic gauge fields to the quasi-particles^14–26^. As a result, the eigen-energies of bulk states become quantized, leading to the Landau levels. Associated phenomena such as flat bands and chiral Landau levels have been experimentally detected in photonic and phononic systems^15,16,20,22–26^.

Different from topological surface states, the chiral Landau level is a one-way propagative bulk state^12,20,25,27^, which is also topologically protected. In quantum field theory and condensed matter physics, chiral Landau levels play an important role in breaking chiral symmetry and resultantly induce the chiral anomaly, characterized by the non-conservation of chiral currents in particle physics and condensed matter physics^27–29^. Prior works on realizing the chiral Landau levels are commonly based on 3D Weyl degeneracies and background magnetic fields^20–22,25,26,28,29^. Compared with 3D Weyl systems, 2D Dirac systems are more accessible for fabrication, and thus are more promising for future applications. Although the theoretical approach for realizing chiral Landau levels in 2D Dirac systems has been proposed^30^, a realistic structure has not been provided and realized in practice. In addition, the linear dispersion relations, and the transport properties of the zeroth chiral mode have not yet been experimentally observed.

In this work, we propose the experimental realization of chiral Landau levels using a photonic honeycomb system. By breaking local parity-inversion symmetry in each unit cell, an inhomogeneous effective mass is introduced into the Dirac cone, which is equivalent to an in-plane synthetic magnetic field coupled with the Dirac quasi-particles. As a result, the energy levels become quantized, and in-plane chiral Landau levels arise, which are one-way propagative and robust against perturbations in the bulk. With the inhomogeneous platform, the band dispersions of Landau levels are experimentally measured. In addition, the robustness of transport of the zeroth chiral mode is also experimentally tested by introducing disordered defects to the system.

## Results

The linear crossings of two energy bands in 2D systems can always be described by the Dirac Hamiltonian ${H}_{D}=v({k}_{x}{\sigma }_{1}\pm {k}_{y}{\sigma }_{2})$, where *v* is the group velocity near the Dirac point, *σ*_1,2,3_ are Pauli matrices, and *k*_*x*_ and *k*_*y*_ are the in-plane Block wave vectors relative to the degeneracy point in *x* and *y* directions, respectively. A typical way to introduce synthetic gauge fields on the 2D plane is to tune the position of the degeneracy points in **k**-space^15,16,22–24,26,31,32^. However, the artificial magnetic field generated in this way is perpendicular to the *xy* plane, which results in flat-band Landau levels for such 2D systems. To obtain an in-plane synthetic magnetic field and consequently further induce in-plane dispersive chiral Landau levels, we need to go beyond the conventional methods. Here, we locally break parity-inversion symmetry^29^ to introduce an effective mass *m*. This mass term will lift the Dirac degeneracy and open a bandgap locally (the gap size $\varDelta \omega =2{|m|}$). If the gap size varies with position, one obtains an effective Hamiltonian that describes the interaction between the Dirac quasi-particles and the synthetic gauge field.

1 $${H}^{{\prime} }=v({\hat{k}}_{x}{\sigma }_{1}\pm {\hat{k}}_{y}{\sigma }_{2})+m({\boldsymbol{r}}){\sigma }_{3}$$

**Fig. 1:**
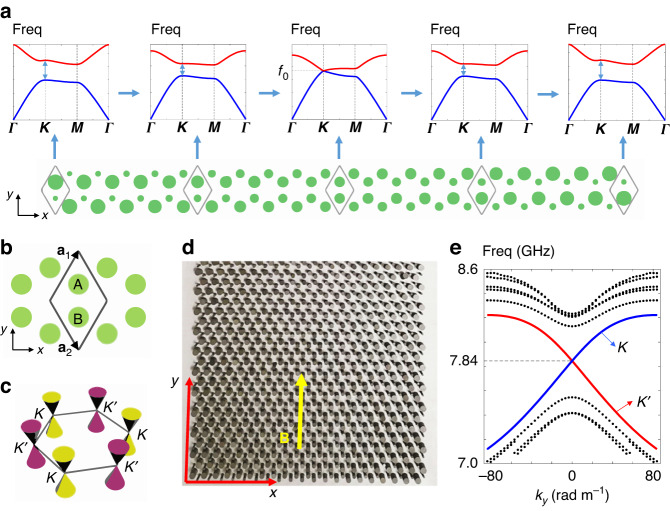
Design of the honeycomb lattice carrying synthetic gauge fields. **a** Design of the supercell honeycomb lattice of the sample. The sample is non-periodic in the *x* direction and periodic in the *y* direction. For each unit cell, local parity-inversion symmetry is broken by tuning the difference of diameter Δ*d* = *d*_*A*_−*d*_*B*_ between cylinders *A* and *B*. Δ*d* varies linearly from positive to negative from the left to the right of the sample. As a consequence, the bandgap Δ*f* at *K* (and *K’*) point varies linearly (approximately) as indicated by the band structures corresponding to the local structure. **b** Unit cell structure of the honeycomb lattice, with A and B being lattice sites made of cylinders with a high dielectric constant (*ε*_m_ = 11.8, estimated value). The unit cell is indicated by the black rhombus, with a_1_ and a_2_ being the lattice vectors. **c** Brillouin zone of the honeycomb lattice, and the band dispersions form Dirac cones near *K* or *K’* pints. **d** A photograph of part of the sample, the inhomogeneous effective mass is equivalent to an artificial magnetic field B in the *y* direction (labeled by the yellow arrow). **e** Full wave simulation of the band structure of the supercell structure under periodic boundary condition in the *y* direction, with dotted lines representing higher order modes, and the blue and red lines are chiral zeroth modes affiliated to *K* and *K’* Dirac points, respectively. The numerical results in the figure are obtained by COMSOL Multiphysics

We realize the model Hamiltonian with the non-periodic system illustrated in Fig. 1a, with the unit cell being the honeycomb structure as shown in Fig. 1b. The unperturbed system has both parity-inversion and time-reversal symmetries (see Fig. 1b), protecting the existence of Dirac cones at the Brillouin zone corners where the energy bands cross linearly at the *K* and *K’* points (see Fig. 1c). Here, the lattice sites A and B are cylinders with a high dielectric constant (*ε*_m_ = 11.8, estimated), and the diameters are denoted by *d*_*A*_ and *d*_*B*_, respectively. The other parts are filled with air (*ε*_air_ = 1). The whole structure (Fig. 1a) is non-periodic in the *x* direction, in which *d*_*A*_ varies from 2 to 6 mm from the left to the right of the sample (*x* direction), and conversely *d*_*B*_ varies from 6 to 2 mm. The periodic boundary condition is applied in the *y* direction. The sign $\pm$ in Eq. (1) corresponds to *K* and *K’* valleys, respectively. ${\hat{k}}_{x,y}$ with hats denote the wave vector operators, and in the direction without translational symmetry, ${\hat{k}}_{x}=-i{\partial }_{x}$ is not a good quantum number. Conversely, in the other direction, the translational symmetry is preserved, and thus ${\hat{k}}_{y}={k}_{y}$ is still a good quantum number. The variation step size between the same lattice sites is fixed at Δ*d*_*A*_ = 0.1 mm (and Δ*d*_*B*_ = −0.1 mm), and thus *d*_*A*_ = *d*_*B*_ = 4 mm on the line *x* = 0 of the sample. This graded morphology causes the gap size Δ*ω* to be linearly dependent (approximately) on the coordinate *x* (as shown in Fig. 1a), which is equivalent to adding an effective mass term to the Dirac Hamiltonian that is linear with respect to *x* ($m={ax}$), and thus Eq. (1) is realized. The photo of part of the fabricated sample for experimental measurement is displayed in Fig. 1d. The 2D Dirac Hamiltonian can be regarded as a subsystem of a synthetic Weyl system ${H}_{W}=v\left({k}_{x}{\sigma }_{1}\pm {k}_{y}{\sigma }_{2}\right)+\widetilde{k}{\sigma }_{3}$ at $\widetilde{k}=0$, where $\widetilde{k}$ is a virtual wave vector used for constructing the Weyl Hamiltonian, and commutes with *k*_*x*_ and *k*_*y*_ (i.e., [*k*_*x*_, $\widetilde{k}$]=0, [*k*_*y*_, $\widetilde{k}$]=0). Since the effective mass also introduces a *σ*_3_ term in the Hamiltonian [Eq. (1)], it is equivalent to a vector potential in the virtual direction [*A* = *m*(*x*)], meaning that an effective canonical momentum operator $\hat{k}=\widetilde{k}+A$ is introduced into the system. The virtual wave vector $\widetilde{k}$ should be distinguished from the physical *k*_*z*_ wave vector that exists in *z*-invariant systems (more discussions are shown in Section 3 of Supplementary Information). Therefore, we expect to see the phenomenon that arises from a synthetic magnetic field in the *y* direction (as indicated by the yellow arrow in Fig. 1d), which is determined by the definition of the magnetic field, i.e., the commutation between canonical momenta ${B}_{y}=[\hat{k},{\hat{k}}_{x}]$.

The designed sample preserves translational symmetry in the *y* direction, and the synthetic magnetic field is also along the *y* direction. In the literature concerned with chiral anomaly (also called axial anomaly), this direction is called the axial direction^26^. The synthetic gauge field will result in the quantization of energy levels, which are expressed as (see Section 1 of Supplementary Information for derivation details).

2 $${\omega }_{n}=\left\{\begin{array}{c} \chi {\mathrm{sgn}}({B}_{y}) v{k}_{y},{n}=0\\ \pm \sqrt{{v}^{2}{k}_{y}^{2}+2n{\rm{|}}a{\rm{|}}v},n\ge 1\end{array}\right.$$

**Fig. 2:**
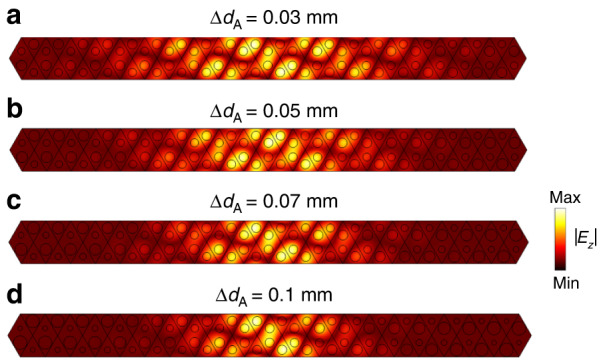
Field confinement (distribution of |*E*_*z*_| in a supercell) tuned by the strength of synthetic magnetic field |*B*_*y*_| which depends on the gradients Δ*d*_A_. **a**–**d** correspond to Δ*d*_A_ = 0.03 mm, 0.05 mm, 0.07 mm and 0.1 mm, respectively. The results are obtained numerically with COMSOL Multiphysics

Here ${\mathrm{sgn}}({B}_{y})$ is the sign of the synthetic magnetic field, and here it is positive because *a* > 0 for our sample. The chirality *χ* can be $\pm$ correspond to the modes in the *K* and *K’* valleys (see Section 2 in Supplementary Information), respectively. From this equation, we see that the energy levels are dispersive in the *k*_*y*_ direction (the direction preserves translational symmetry) but independent of the virtual wave vector $\widetilde{k}$. The zeroth-order Landau level has a linear dispersion and can have group velocities in +*y* (up-going) or −*y* (down-going) directions, which is determined by its projection to the *K* or *K’* valleys, i.e., the chirality of the Dirac point. For the experimental sample, the band structure of the supercell under periodic boundary conditions in the *y* direction (Fig. 1a) can be interpreted as the Landau levels under the action of a synthetic gauge field, and the full wave simulation result (near *k*_*y*_ = 0, the blue dot) is plotted in Fig. 1e. Quantized energy levels can be conspicuously observed from the figure, with the dotted lines show high order Landau levels, and the linearly dispersive zeroth modes are labeled by blue and red lines, which are affiliated to *K* and *K’* valleys, respectively. The analytical expressions of Landau levels (Eq. 2) can well reproduce the salient features of the band structure if the *k*_*y*_ domain is in the vicinity of the *K* or *K’* points (see Section 2 of Supplementary Information for details). Our results show that mini gaps exist between any two adjacent Landau levels in our 2D system (see Fig. 1e). As predicted by Eq. (2), the gap width can be tuned by the strength of the synthetic magnetic field *B*_*y*_ (equivalent to tuning the gradient Δ*d*_*A*_ or |*a*|). Increasing |*a*| (or Δ*d*_*A*_) will result in a larger gap width between adjacent energy levels (e.g., the gap between 0th and 1st order levels $\varDelta {\omega }_{1}=\sqrt{2|a|v}$), and conversely, the gap width decreases by decreasing |*a*| (or Δ*d*_*A*_). More numerical results on the gap width and the strength of the synthetic magnetic field are shown in Section 4 of Supplementary Information. An interesting feature can be deduced from Eq. (2) that the group velocity ($\partial \omega /\partial {k}_{y}$) of the zeroth mode is independent of the strength of the synthetic magnetic field (i.e., |*a*|), meaning that increasing or decreasing the gradient (i.e., the value of Δ*d*_*A*_ or Δ*d*_*B*_) will not change the slope of the linear dispersion. More numerical results substantiating the relation between the group velocity of zeroth mode and the gradient |*a*| are also shown in Section 4 of Supplementary Information. The ability to control the field confinement of eigenstates plays a useful role in many applications, including sensing devices^32,33^. For the Landau levels in our designed system, the field confinement depends on and hence can be controlled by the strength of the synthetic magnetic field. We take the zeroth mode at *k*_*y*_ = 0 as an example and choose four different gradients Δ*d*_*A*_ representing different strengths of synthetic magnetic fields *B*_*y*_. Numerical results are shown in Fig. 2, with Fig. 2a–d corresponding to Δ*d*_*A*_ = 0.03 mm, 0.05 mm, 0.07 mm and 0.1 mm, respectively. We see that the high field intensity region decreases as the gradient Δ*d*_*A*_ increases, meaning that increasing the strength of synthetic magnetic field *B*_*y*_ can lead to stronger field confinement. It is notable that the boundary condition in the *x* direction does not affect the computed dispersions, because the field of the eigenstates in the frequency range of interest is confined near *x* = 0 (see Fig. 2), owing to the fact that the virtual wave vector $\widetilde{k}$ is zero in our system. Even though the Landau level dispersions are independent of $\widetilde{k}$, the localization of eigenstates is $\widetilde{k}$-dependent, which is always confined to the vicinity of the area that satisfies $\widetilde{k}+A=0$. We note that the Landau levels arising from synthetic gauge fields always pertain to a specific gauge.

**Fig. 3:**
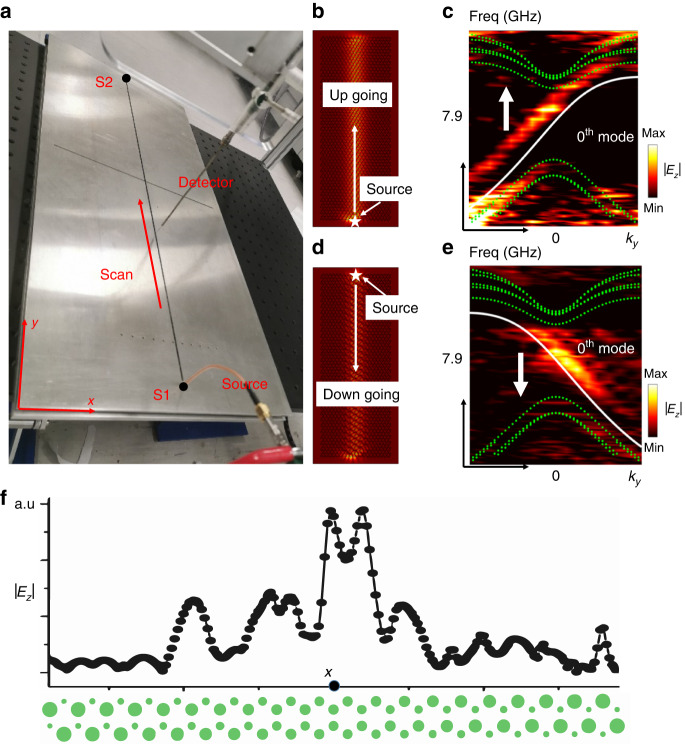
Experimental measurement of chiral Landau levels. **a** A picture of the experimental setup. The source and the detector are connected to ports of the vector network analyzer. The whole sample is covered with a metal plate, and the detector will be inserted into the plate to scan the near field along the arrow. The point source with an out-of-plane polarization can be placed at the bottom center (position S1) or at the top center (position S2). **b** Field strength |*E*_*z*_| distribution (simulation result) excited by the point source at position S1. **c** Dispersion of Landau levels obtained by experimental measurement with the excitation source located at S1. The up-going chiral zeroth mode is excited. **d** Field strength |*E*_*z*_| distribution (simulation result) excited by the point source at position S2. **e** Dispersion of Landau levels obtained by experimental measurement with the excitation source located at S2. The numerical results are displayed with solid (zeroth mode) and dotted lines (higher order modes) in (**c**) and (**e**). **f** Field strength distribution along *x* direction (|*E*_*z*_|) of zeroth-order chiral Landau level obtained from experimental measurement

To experimentally measure the Landau levels, we scan the near field with our experimental setup (Fig. 3a). To ensure the precision in experimental measurement (see “Materials and methods” for details), we cover the sample with a metal plate, on which a slit is etched in the middle so that the detector can insert in to measure the near field. We first locate the point source at the bottom center (position S1), and scan the near field along the slit indicated by the arrow (Fig. 3a). The up-going zeroth mode can be excited as indicated by the numerical result in Fig. 3b. Using a discrete Fourier transformation, the energy level dispersions can be obtained experimentally, and the Fourier transformed field intensity |*E*_*z*_| as a function of *k*_*y*_ and *f* is shown in Fig. 3c. The zeroth mode can be observed, which agree well with solid blue line obtained with the full wave simulations, respectively. The result is also consistent with the theoretical predictions (see Supplementary Fig. S1). The central frequency deviates slightly from the simulation results as indicated by the figure because the experimental sample is a slab with finite thickness instead of a *z*-invariant system (see details in “Materials and methods”). We see that for the zeroth-order Landau level, only the upward (+*y*) propagating mode is excited if the source is located at the bottom center. The dispersion is linear, as expected. By contrast, if we put the source at the top center (position S2 in Fig. 3a), only the zeroth mode that is propagating in the −*y* direction can be excited (as indicated by the field distribution in Fig. 3d). This is further confirmed via energy level dispersions in Fig. 3e obtained from a discrete Fourier transformation, where the zeroth mode only has a negative group velocity. The field distribution |*E*_*z*_| of the zeroth-order chiral mode in *x* direction is also measured, as shown in Fig. 3f. It is seen that the mode amplitude is localized near the middle (*x* = 0) of the sample, consistent with our previous discussions (i.e., due to $\widetilde{k}=0$). Since the up-going and the down-going zeroth chiral mode are affiliated to *K* and *K’* valleys respectively, the electromagnetic response of *K* and *K’* points are different from each other if the excitation frequency is within the bandgap between ±1st order Landau levels. Relevant discussions are shown in Section 2 of Supplementary Information. The observed phenomenon complies with the symmetry of the system. Even though the local parity-inversion symmetry of each unit cell is broken, the overall sample still has a parity-inversion symmetry. The sources at the bottom center and the top center can be transformed to each other via parity-inversion operations. Correspondingly, and the positively and negatively propagating chiral modes excited by the two sources (as well as *K* and *K’* points) are also parity-symmetric to each other. We note that the overall system preserves time-reversal symmetry, and the up-going and the down-going zeroth modes, affiliated to *K* and *K’* valleys, form a time-reversal symmetric pair. However, if one considers the transport for states residing in one single valley, the single zeroth mode is one-way propagative. Such a consideration is reasonable because inter-valley scattering is usually small.

**Fig. 4:**
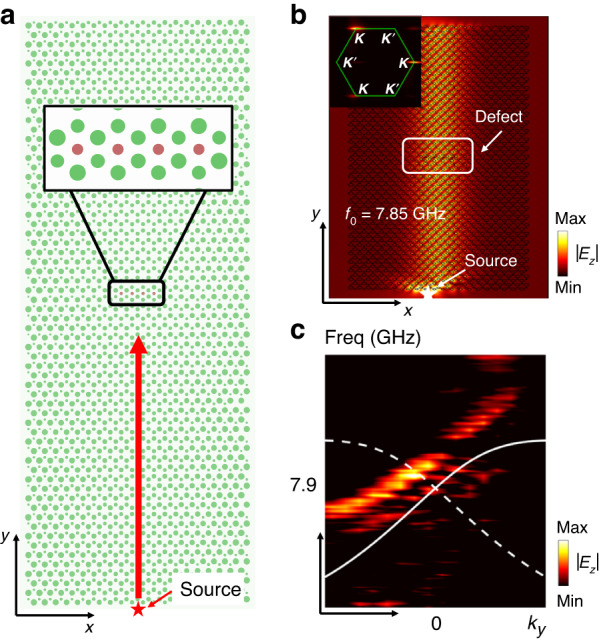
Experimental test on the transport robustness of the chiral zeroth Landau level. **a** Putting a defect composed of dielectric cylinders at the center of the sample, which spans almost the domain of the field distribution (*x* direction) of the zeroth mode to disrupt its propagation. A point source is placed at the bottom center of the sample. Inset: the zoomed-in picture of the defect. **b** Full wave simulation of the magnitude of the field (|*E*_*z*_|) distribution (at frequency 7.85 GHz, inside the gap between ±1st Landau levels) under the excitation condition shown in panel (**a**). Weak field localization can be observed within the defect domain, but the reflection is almost negligible because no interference pattern can be observed. **c** Experimental result of Landau level dispersions resolved from the field distributions with defect introduced (see panel (**a**)). The up-going zeroth mode (solid blue line) is dominantly excited, and the excitation of the down-going mode (dash blue line) due to reflection at the defect is negligible. The solid and dashed lines represent numerical results

The chiral zeroth Landau level is robust against small perturbations. Next, we provide a more detailed experimental test for the robustness of transport against imperfections. We first put a point source at the center of the bottom edge of the sample, and at the same time, some defects (4 additional cylinders with a diameter of 3 mm) are introduced at the center of the sample, as shown in Fig. 4a. The zoomed in picture of the defects is shown in the inset. Compared with Fig. 3f, we find that the defects almost block the entire confined region of the field of the zeroth mode, and such defects will result in strong reflections for ordinary propagating modes. However, from the simulation result in Fig. 4b, we find that even though weak localization of the field strength distribution (|*E*_*z*_|) can be observed at the center of the sample (location of the defect), the chiral zeroth mode is not impeded by the defect. The reflected field intensity of the down-going zeroth mode is negligibly small compared with the transmitted field intensity, evidenced by the fact that no interference pattern can be observed. We also performed the 2D Fourier transformation of the field distributions, and the modes at *K’* valley (down-going zeroth mode) are almost not excited compared with those at *K* valley (up-going zeroth mode), even though the defect is introduced (see the inset of Fig. 4b). We next measure the dispersion of the Landau levels to quantitatively test the reflection strength. By locating the point source at the bottom center, the field distribution (|*E*_*z*_|) in *k*_*y*_-*f* space can be resolved experimentally. It is shown that the zeroth mode that is propagating in the −*y* direction is almost not excited (dashed blue line) compared with that in the +*y* direction (solid blue line), showing the negligible impact from the defect, as indicated by the dispersions in Fig. 4c. The experimental results provide solid evidence for the robustness of the transport of the chiral zeroth Landau level. In Section 5 of the Supplementary Information, we introduce a much larger defect to test the robustness of the zeroth mode, and the same conclusion can be reached by observing the results in Supplementary Fig. S5. The weak backscattering of the chiral zeroth Landau level can be intuitively understood. Since the zeroth modes with positive and negative group velocities are affiliated to different valleys (i.e., *K* and *K’*), the backscattering of the zeroth mode is essentially an inter-valley scattering if the illumination frequency is inside the gap. Since the *K* and *K’* points are widely separated in **k**-space, the inter-valley coupling is weak.

## Discussion

To summarize, we propose a realistic system to realize chiral zeroth Landau levels via inhomogeneously breaking local parity-inversion symmetry in 2D Dirac point systems. Such a system is experimentally realized using a photonic honeycomb lattice system, in which the diameter of lattice sites in each unit cell is tuned to introduce an effective mass that depends linearly on the spatial coordinate. Based on such a platform, the in-plane chiral Landau level dispersions are experimentally measured. The robustness of the zeroth-order mode is experimentally demonstrated by introducing defects at the center of the sample. Our proposal extends the realization of chiral zeroth Landau level from 3D systems to 2D systems, which are much easier to fabricate, and are hence more promising for applications using chiral response^34–38^. By scaling the sample from the millimeter region to the nanometer region, the scheme may inspire the design of low-loss photonic crystal components that utilize the robustness of the chiral Landau levels.

## Materials and methods

The sample we fabricated is composed of cylinders with a high refractive index, which are placed at the lattice sites of a honeycomb lattice. The cylinders are made of yttrium iron garnet with a dielectric constant of 11.8 (estimated), and all have the same height (*h* = 10 mm). To ensure the mechanical stability of the system, we fabricated a metal plate, on which round holes are etched with appropriate diameters so that the cylinders can stand steadily. The holes are etched with a thickness of 2 mm, and thus most parts of the cylinders (8 mm) are outside of the hole and surrounded by air. We then cover the sample with another metal plate, the part between the two metal plates can well reproduce the designed 2D system. The only difference is that the realistic sample is a 3D system because the thickness is finite (8 mm). The perfect electric conductor (PEC) boundary condition imposed by the two metal plates ensures that the model profile is uniform along the *z*-direction so that the band structure is consistent with the ideal 2D system. The finite thickness of the sample only induces a shift of central frequency from 7.84 to 7.9 GHz. The parity-inversion symmetry is not perfect in the experimental sample, which originates from the fabrication errors. This will induce the difference of central frequencies of the up-going and the down-going zeroth modes, which can be indicated by Fig. 3c, e.

The experimental setup is made of a 3D translational platform, equipped with a network analyzer with two ports, one of which acts as a point source to generate electromagnetic waves and the other acts as the detector. The eigen-modes we measured have the out-of-plane electric field vector (*E*_*z*_), and thus the detector antenna should be perpendicular to the plate so that the impact from other modes with in-plane electric vector (*E*_*x,y*_) can be excluded (as shown in Fig. 3a, c). In measuring the electric field (*E*_*z*_) distribution, the detector antenna should be placed within the air region between the metal plates. The Landau level is a 1D dispersion so we only need to measure the field distribution along a line parallel to the *y*-axis. We thus etched a slit in the middle of the upper plate (as shown in Fig. 3a, c) through which the detector antenna can be inserted. We note that the slit should be thin enough so that the PEC boundary condition almost remains intact. A thick slit could introduce more noise to the measured result. The detector antenna cannot be inserted into the cylinders, which makes the 2D measurement more difficult. In Supplementary Fig. S1e, f, the field distribution beyond the first Brillouin zone in **k**-space is also needed. Therefore, the field of more points within each unit cell is required to obtain the result. As a consequence, we have to measure the field inside the cylinders (so that we have enough grid points to do a Fourier transform), which cannot be accomplished by the current setup. We therefore only provide the numerical result of 2D **k**-space field distribution (Supplementary Fig. S1e, f), and the experimental result cannot be provided.

## Supplementary information

SUPPLEMENTAL MATERIAL

## Data Availability

All data are available in the main text and the supplementary information.

## Code availability

All codes are available in the main text and the supplementary information.
