# Supplementary material for: Experimental realization of chiral Landau levels in two-dimensional Dirac cone systems with inhomogeneous effective mass
Source: Light Sci Appl. 2023 Jul 4;12:165. doi: 10.1038/s41377-023-01209-z (PMC10319816; doi:10.1038/s41377-023-01209-z)
Supplement: Supplementary file 1 — SUPPLEMENTAL MATERIAL [file 41377_2023_1209_MOESM1_ESM.docx]

Supplementary information for experimental realization of chiral Landau levels in two-dimensional Dirac cone systems with inhomogeneous effective mass

Hongwei Jia^1,2,#^,*, Mudi Wang^1,#^, Shaojie Ma^3^, Ruo-Yang Zhang^1^, Jing Hu^1^, Dongyang Wang^1^, Che Ting Chan^1,†^

^1^Department of Physics, the Hong Kong University of Science and Technology, Clear Water Bay, Kowloon, Hong Kong, China

^2^Institute for Advanced Study, the Hong Kong University of Science and Technology, Clear Water Bay, Kowloon, Hong Kong, China

^3^Department of Physics, University of Hong Kong; Hong Kong, China

^#^these authors contributed equally to this work

Corresponding authors: *[jiahongwei7133@gmail.com](mailto:*jiahongwei7133@gmail.com), ^†^[phchan@ust.hk](mailto:phchan@ust.hk)

Telephone number: *(852) 2358 7980, ^†^(852) 2358 7487

**1. Derivation of Landau level dispersions**

Here we derive the dispersion relation of Eq. (2) in the main text. We start from the Dirac equation coupled with an inhomogeneous effective mass term (Eq. 1 in the main text), in which the effective mass is linear with respect to *x*-direction.

$H'=v(\hat{k}_{x}\sigma_{1}\pm\hat{k}_{y}\sigma_{2})+ax\sigma_{3}$ (S1)

As the Hamiltonian includes Pauli matrices, we first square the Hamiltonian

(S2)

Here we used the following commutation relations

$[\hat{k}_{x},\hat{k}_{y}]=0, [\hat{k}_{y},x]=0$ (S3)

Since $\hat{k}_{x}=-i\partial_{x}$, it is not difficult to find the commutation

$[\hat{k}_{x},x]=-i\partial_{x}x=-i$ (S4)

Based on the commutation relation, we can define the creation and annihilation operators

$b^{\dagger}=N(v\hat{k}_{x}-i|a|x), b=N(v\hat{k}_{x}+i|a|x)$ (S5)

The commutation relation $[b^{\dagger},b]=1$ requires that

$\begin{matrix} 1=[N(v\hat{k}_{x}-i|a|x),N(v\hat{k}_{x}+i|a|x)]=N^{2}([v\hat{k}_{x},i|a|x]+[-i|a|x,v\hat{k}_{x}]) \\ =2iv|a|N^{2}[\hat{k}_{x},x]=2N^{2}v|a| \\ \Rightarrow N=\frac{1}{\sqrt{2v|a|}} \end{matrix}$ (S6)

Next we define the particle number operator

$\begin{matrix} \hat{n}=b^{\dagger}b=N^{2}(v\hat{k}_{x}-i|a|x)(v\hat{k}_{x}+i|a|x) \\ =N^{2}(v^{2}\hat{k}_{x}^{2}+a^{2}x^{2}+[v\hat{k}_{x},i|a|x])=N^{2}(v^{2}\hat{k}_{x}^{2}+a^{2}x^{2}+v|a|) \end{matrix}$ (S7)

and we thus have

$\frac{1}{N^{2}}\hat{n}=v^{2}\hat{k}_{x}^{2}+a^{2}x^{2}+|a|v$ (S8)

Eq. (S2) reduces to

(S9)

There is still a $\sigma_{2}$ term in Eq. (S9), and we need the following process

(S10)

It is then not difficult to derive the analytical expression of Landau levels

$\omega_{n}=\left\{\begin{matrix} \pm\sqrt{v^{2}k_{y}^{2}+2n|a|v} n\geq1 \\ \chi vk_{y} n=0 \end{matrix}\right.$ (S11)

The Landau level dispersions in Eq. (2) of the main text are thus obtained.

**2. Numerical simulations**

In calculating the Landau levels of the Dirac points with inhomogeneous effective mass, the *k_y_* component of the Block wave vector should be in the vicinity of the *K* or *K'* points. This means that Eq. (S11) is valid only if *k_y_* is near the *Γ* points (i.e. *k_y_* is near $2n\pi/\sqrt{3}a$, ) in the **k**-space of the supercell system. However, the complete band structure of the supercell under periodic boundary condition in *y* direction can be numerically obtained. In Fig. S1a (also Fig. 1e in the main text), we show the simulation results of the band structure calculated by the wave optics module of COMSOL. Landau level dispersions can be easily identified from the figure. It is also found that zeroth modes with both positive and negative group velocities are in the band structure, because the results include the Landau levels of all the valleys (both *K* and *K'* points). Each of them is affiliated to a *K* or a *K'* point in the Brillouin zone of the 2D honeycomb lattice (the inset of Fig. S1a), as specified in the figure. For comparison, we also show the theoretical results of Landau level dispersions obtained by Eq. S11, as displayed in Fig. S1(b-c) for *K* and *K'*, respectively. As expected, the analytical expression can well reproduce profile of the band dispersion if *k_y_* is near $2n\pi/\sqrt{3}a$, but derivate from the full wave simulation results if *k_y_* is away from $2n\pi/\sqrt{3}a$. This is because the Dirac Hamiltonian can only describe the cone-like dispersions near *K* or *K'* points.

In the main text, the Landau level dispersions are experimentally measured under different excitation conditions. Within the bandgap between ±1st order Landau levels, the electromagnetic response of *K* and *K'* valley are different from each other, owing to the fact that the zeroth mode is either up-going or down-going and affiliated to different valleys (see Fig. S1d). Therefore, if the source is located at the bottom center to excite the up-going zeroth mode (see Fig. 2a-b), only the eigenstates at *K* point can be excited. As indicated in Fig. S1e, the field strength |*E_z_*| at *K* points is much higher than that at *K'* at any frequency inside the gap (here we set the frequency to the central frequency). Conversely, if the source is located at the top center to excite the down-going chiral zeroth mode, only the eigenstates at *K'* points can be excited, and resultantly, the field strength |*E_z_*| at *K'* point is much higher than that at *K* point (see Fig. S1f). The results in Fig. S1e-f are obtained numerically.

The honeycomb lattice is a typical 2D system, with its structure homogeneous in *z* direction and extends to infinity. However, such a structure cannot be realized in experiment, and thus mostly we truncate the structure and apply perfect electric conductor (PEC) boundary condition in *z* direction, so that the structure can have a finite size. Such a truncation of the sample does not change profile of the band dispersions because the symmetries of the structure remain unchanged. However, the frequency will be shifted a little. In our system, the central frequency obtained numerically is *f*_0_=7.837 GHz, which is shifted to *f*_0_=8GHz in experiment.

**3. The impact of out of plane wave vector in the z-invariant 2D photonic crystal**

A *z*-invariant system corresponds to a 2D photonic crystal that is infinite in the *z*-direction, which cannot be fabricated. In the realistic system, the thickness is finite and *k_z_* is not well defined. The experimental sample is a 2D subsystem of a 3D system, in the sense that the thickness of the sample will not change the 2D space group symmetry, and thus degeneracy features remain the same as a true 2D system. In the main text, we showed that we can mathematically attach a 3^rd^ dimension to the 2D system, which has a corresponding virtual wave vector $\tilde{k}$, allowing us to introduce the term $\tilde{k}\sigma_{3}$ to the Dirac Hamiltonian. This synthetic dimension allows for a Weyl Hamiltonian to be constructed, and thus the artificially generated magnetic field $B_{y}=[\hat{k}_{x},\hat{k}]$ can be defined. This synthetic wave vector $\tilde{k}$ should be distinguished from the physical *k_z_* wave vector in the *z*-invariant 2D photonic crystal. In this section, we discuss what will happen if we consider a *z*-invariant photonic crystal (rather than a thin photonic slab).

Put into the context of a *z*-invariant 2D photonic crystal, we consider transverse electric modes (TE) with wave vector *k_z_* is zero, which only have out of plane electric vector (*E_z_*). If we consider modes with wave vector *k_z_* not equal to zero, the modes will have in-plane electric vectors (*E_x_* and *E_y_*). The nonzero *k_z_* will not introduce additional effective mass to the Dirac Hamiltonian, and only the central frequency is slightly shifted. Therefore, in the inhomogeneous system with synthetic magnetic field, the frequency of Landau levels will also be shifted if we change the *k_z_* wave vector from zero to a non-zero value. In Fig. S2, we provide the numerical result of Landau levels for different *k_z_* in the *z*-invariant photonic crystal. As can be indicated, the central frequency *f*_0_ is shift from 7.837 GHz to 7.924 GHz by changing *k_z_*=0 to *k_z_*=30.

The Landau level dispersions are independent of $\tilde{k}$ as indicated by Eq. (2) in the main text. However, the field distribution of Landau levels depends on $\tilde{k}$, which is always confined near by the position satisfying $\tilde{k}+A(x)=0$. Conversely, in the *z*-invariant photonic crystal, the dispersion depends on the *k_z_* wave vector (indicated by Fig. S2), but the localized area of Landau mode is independent of *k_z_*. As indicated by Fig. S3, the field is always localized nearby the center (*x*=0) of the system even though we change *k_z_* from 0 to 30 rad m^-1^.

**4. Gap size between adjacent levels, group velocity of zeroth mode and strength of synthetic magnetic field**

In the main text, we provided a brief introduction on the dependence of gap width and group velocity of zeroth mode on the strength of synthetic magnetic field. Here we provide more details on this relationship. The strength can be directly related to the gradient of the structure Δ*d*_A_. We first plot the numerical results of the dispersion of Landau levels for different Δ*d*_A_ in Fig. S4, where (**a**-**d**) correspond to Δ*d*_A_=0.03, 0.05, 0.07 and 0.1 mm, respectively. To better present the result, we extract the data of Δ*ω*_1_ (between the zeroth order and the first order Landau levels), Δ*ω*_2_ (between the zeroth order and the first order Landau levels) and vg (the group velocity, or the slope of the linear dispersion of the zeroth mode) for different Δ*d*_A_ into Table S1. Obviously, both gap widths Δ*ω*_1_ and Δ*ω*_2_ are increasing with the increase of the gradient Δ*d*_A_ (or the strength of synthetic magnetic field). Conversely, *v_g_* stays unchanged as we change Δ*d*_A_. These results can be well predicted with the dispersion of the Landau levels [i.e. Eq. (2) in the main text].

**5. Transport robustness of the zeroth mode against a large defect**

In the main text, we experimentally tested the transport robustness of the zeroth chiral Landau level by introducing a defect into the bulk. The experimental result (Fig. 4c) shows that the zeroth mode propagating in the -*y* direction was almost not excited even though the defect is introduced, indicating the weak backscattering of the zeroth mode. Here we provide the numerical result of Fig. 4c to avoid uncertainty in the experimental, and result is plotted in Fig. S5a. This plot is clearer to show that the down-going zeroth mode is almost not excited compared with the up-going zeroth mode, attributed to the weak scattering at the defect.

Next, we introduce a much larger defect, as shown by the inset of the left panel of Fig. S5b. We first put the point source at the bottom center and the excited field distribution is shown in the right panel of Fig. S5b. It is seen that the negative propagating zeroth mode is almost not excited because no interference pattern can be observed at frequency 7.85 GHz (inside the gap between ±1^st^ order Landau levels). By resolving the field distribution in the **k**-space (2D Fourier transformation result), we find that only the modes at *K* valley are excited, and the excitation of modes at *K'* valley is negligible (Fig. S5c). We further resolve the Landau level dispersions via a 1D Fourier transformation, and it is found that the negative propagating mode is almost not excited. Therefore, the transport robustness of the zeroth mode is again further tested with a larger defect, and we arrive the same conclusion as that in the main text. The results in Fig. S5 are obtained numerically.


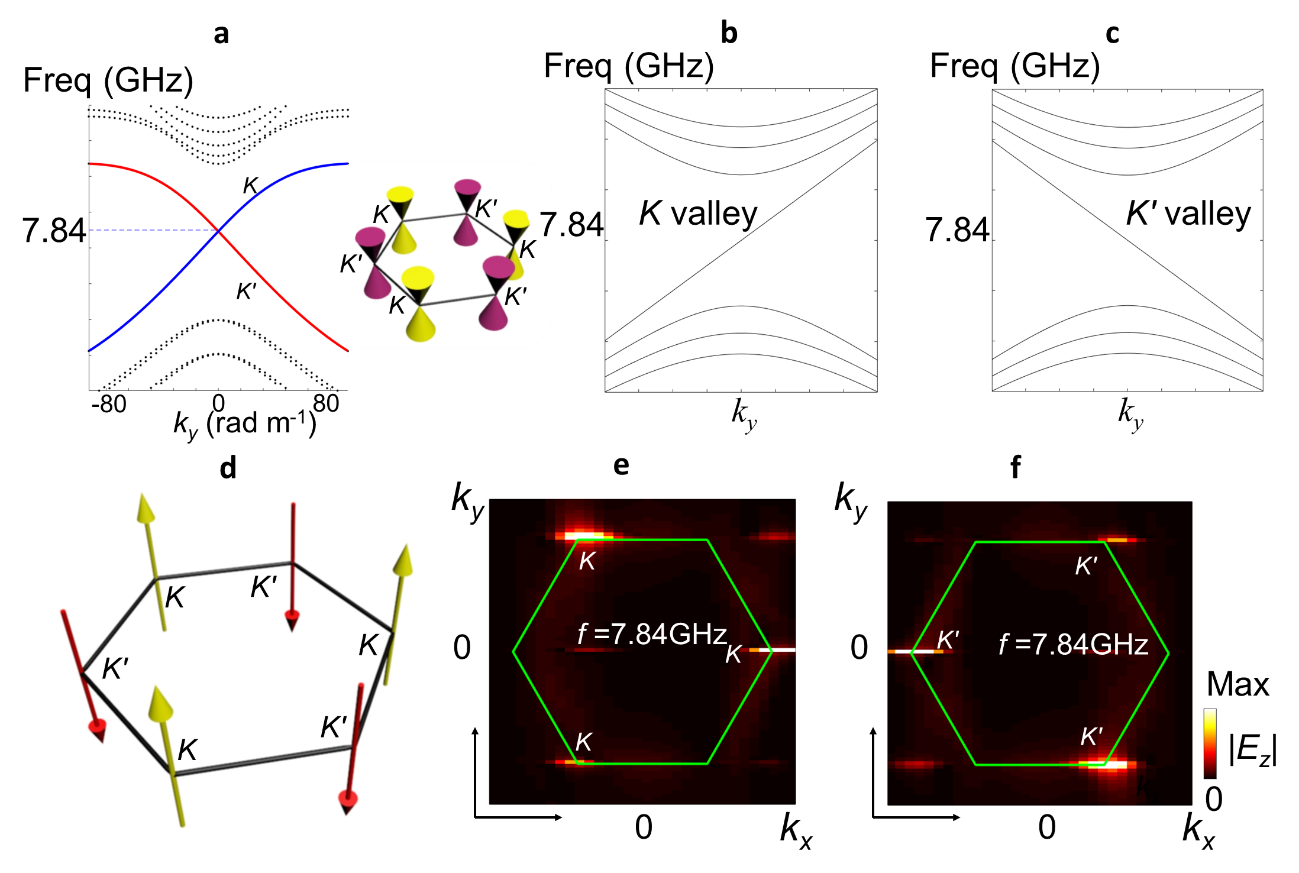


**Fig. S1 Comparison of Landau levels between full wave simulation and analytical expressions and valley dependent chiral transport behavior.** **a** Full wave simulation of the band structure of the supercell system in Fig. 1c under periodic boundary condition in *y* direction. Results are obtained with the wave optics module of COMSOL. The band structure can be interpreted as the Landau levels, with the zeroth modes having a positive group velocity affiliated to *K* valley, and those having a negative group velocity affiliated to *K'* valleys (as specified in the figure). (**b**-**c**) Landau level dispersions obtained by Eq. (S11). The results can well predict the band structures if *k_y_* is near the *K* or *K'* points (i.e. *k_y_* is near $2n\pi/\sqrt{3}a$, , labelled by blue dots), and diverges from the simulation result if *k_y_* is away from $2n\pi/\sqrt{3}a$. **d** Chiral transport of zeroth order Landau levels. The zeroth mode of *K* point is propagative in positive direction, while the zeroth mode of *K'* point is in negative direction. (**e**-**f**) 2D Fourier transformed field distribution for point source locating at the bottom center and top center respectively at central frequency, corresponding to the setup in Fig. 2a and 2c in the main text. When only positively propagating zeroth mode is excited, *K* points have higher field intensity than *K'* points. Conversely, the field at *K'* points is more pronounced.


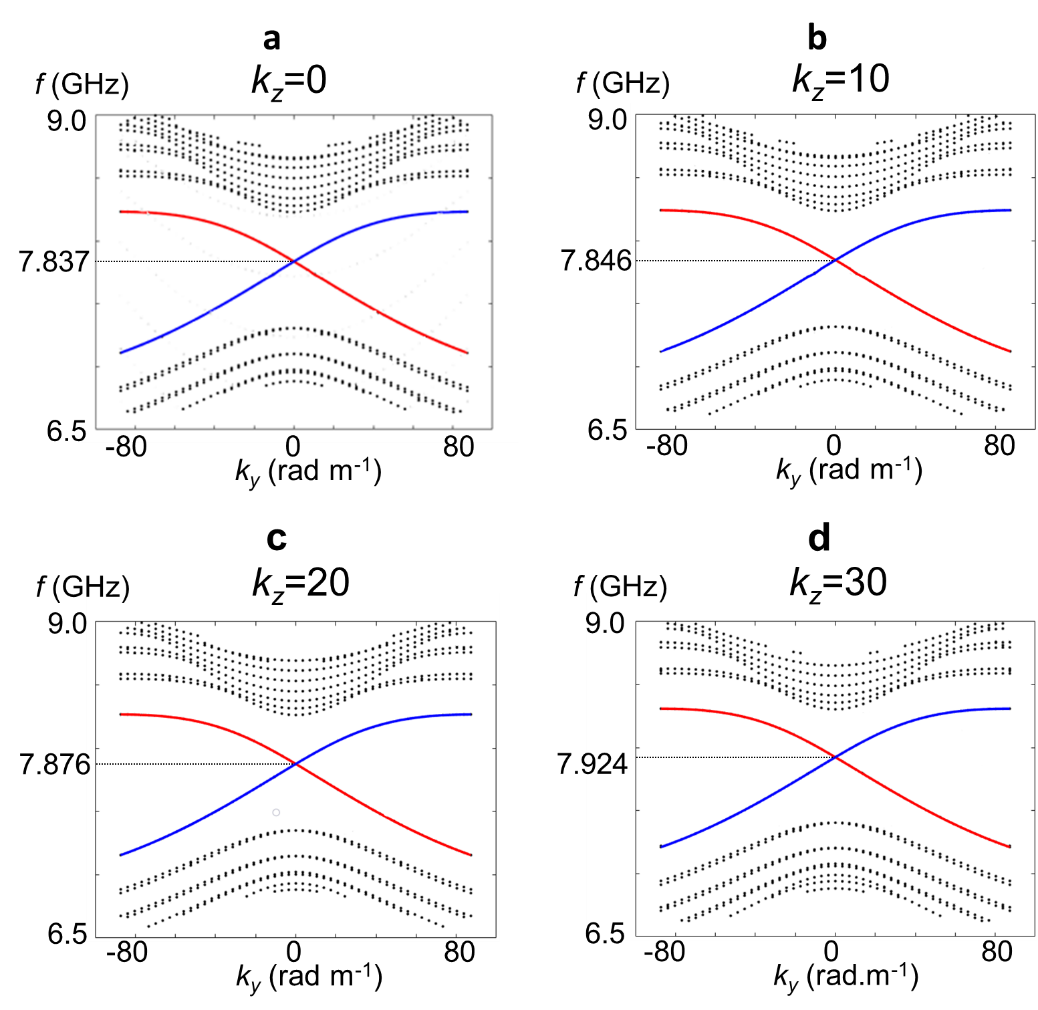


**Fig. S2 Landau level dispersions at different *k_z_* of a *z*-invariant system.** (**a**-**d**) correspond to *k_z_*=0, 10, 20 and 30 rad m^-1^ respectively. Changing *k_z_* only shifts the frequency.


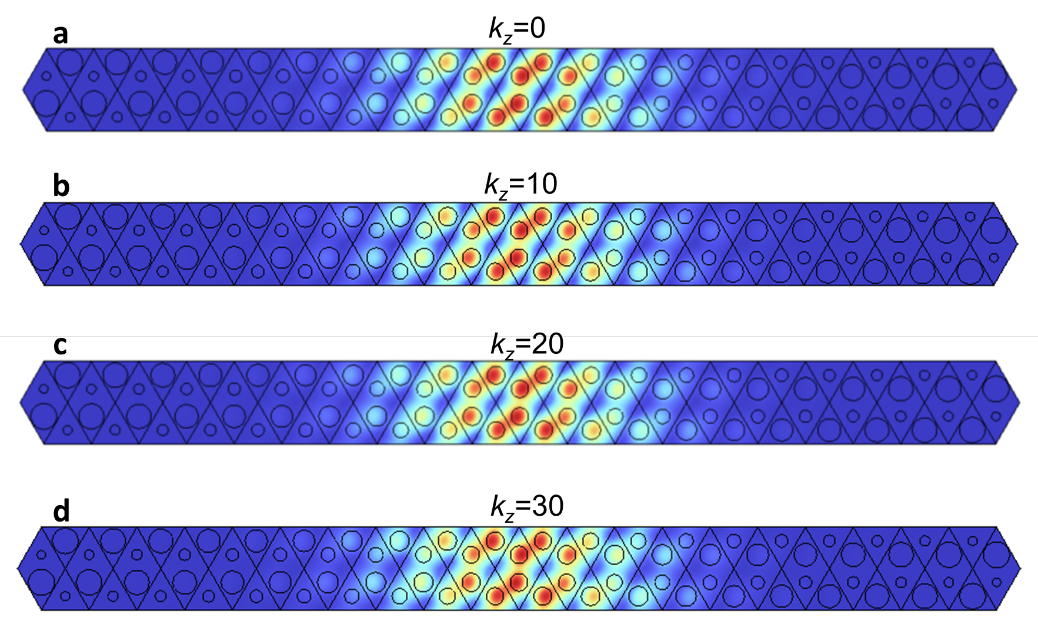


**Fig. S3 Field distribution of the zeroth order Landau level for different *k_z_* in the *z*-invariant ideal system.** (**a**-**d**) correspond to *k_z_*=0, 10, 20 and 30 rad m^-1^, respectively. The field strength is localized in the area corresponding to the zero of the synthetic gauge field and is independent of *k_z_*.


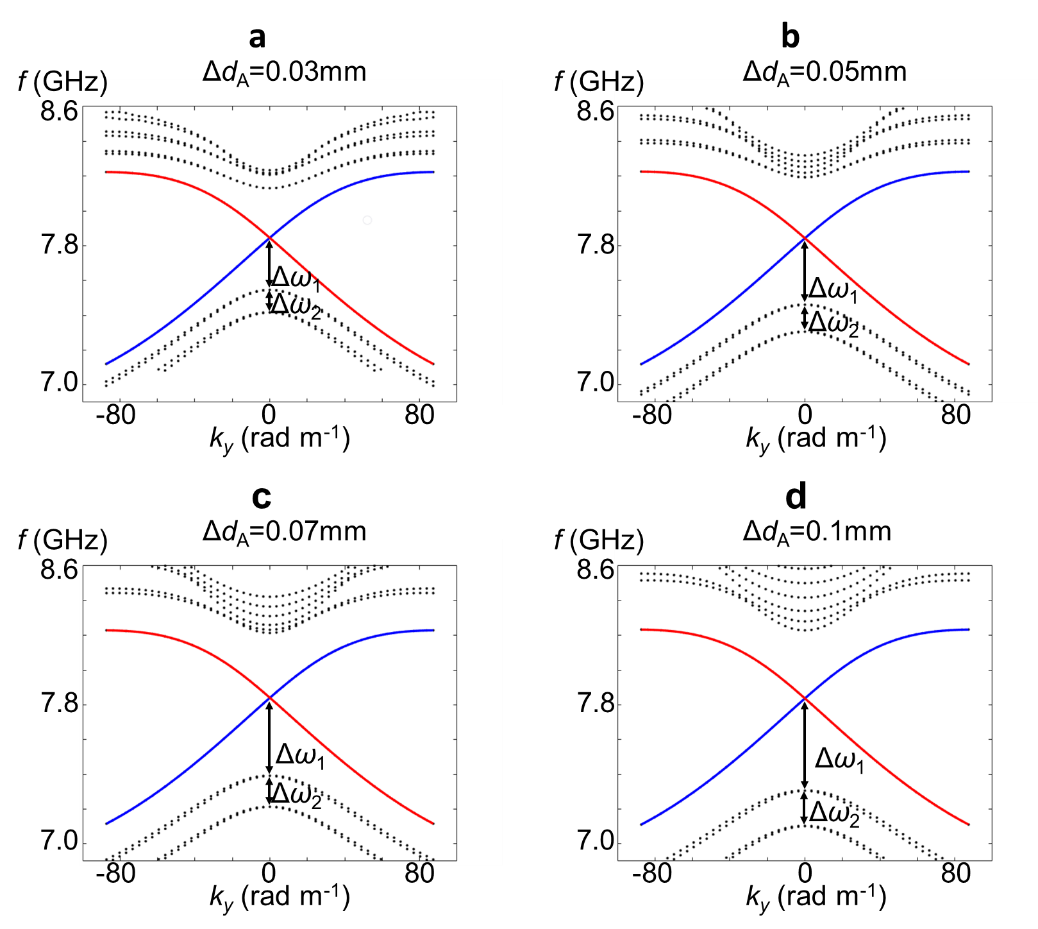


**Fig. S4 Landau level dispersions for different gradients Δ*d*_A_.** (**a**-**d**) correspond to Δ*d*_A_ =0.03, 0.05, 0.07 and 0.1 mm, respectively. The experimental sample is fabricated based on Δ*d*_A_=0.1mm. The gap width between the zeroth order and the first order Landau levels is denoted by Δ*ω*_1_, and the gap width between the first order and the second order Landau levels is denoted by Δ*ω*_2_.

|  | Δ*d*_A_=0.03mm | Δ*d*_A_=0.05mm | Δ*d*_A_=0.07mm | Δ*d*_A_=0.1mm |
| --- | --- | --- | --- | --- |
| Δ*ω*_1_ | 0.299GHz | 0.382GHz | 0.449GHz | 0.531GHz |
| Δ*ω*_2_ | 0.129GHz | 0.155GHz | 0.178GHz | 0.203GHz |
| *v_g_* | 5.85×10^7^m/s | 5.85×10^7^m/s | 5.85×10^7^m/s | 5.80×10^7^m/s |

**Table S1** Gap widths between the zeroth order and the first order Landau levels Δ*ω*_1_, and between the first order and the second order Landau levels Δ*ω*_2_ for different gradients Δ*d*_A_. The group velocity of zeroth order Landau level for different gradients Δ*d*_A_. Δ*d*_A_ can be directly related to the strength of applied synthetic magnetic field *B_y_*. Data are extracted from Fig. S4.


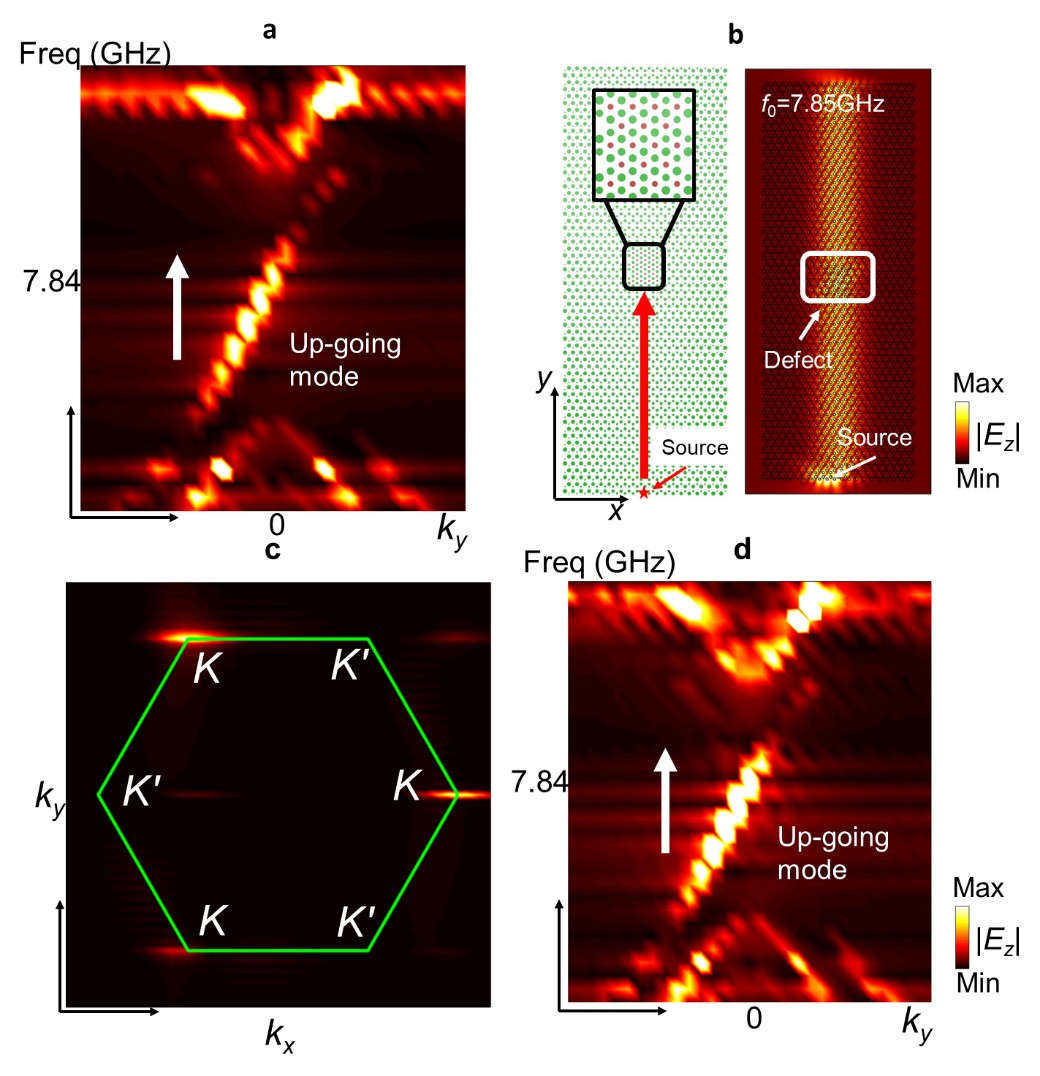


**Fig. S5 Transport robustness of the zeroth mode against a larger defect. a** The corresponding numerical result of Fig. 4c. **b** Left panel: locating the defect at the center of the sample and the point source at the bottom center. Inset: zoomed in picture of the defect. Right panel: field strength |*E_z_*| distribution of the excited field by the point source. **c** 2D Fourier transformation pattern of the field distribution in the right panel of (**b**). The modes at *K* valley are pre-dominantly excited than those at *K'* valley. **c** Dispersion of Landau levels in the presence of the large defect in (**b**). The down-going zeroth mode is almost not excited compared with the up-going zeroth mode. All the results in the figure are obtained numerically.
